# SysBiolPGWAS: simplifying post-GWAS analysis through the use of computational technologies and integration of diverse omics datasets

**DOI:** 10.1093/bioinformatics/btac791

**Published:** 2022-12-08

**Authors:** Oluwadamilare Falola, Yagoub Adam, Olabode Ajayi, Judit Kumuthini, Suraju Adewale, Abayomi Mosaku, Chaimae Samtal, Glory Adebayo, Jerry Emmanuel, Milaine S S Tchamga, Udochukwu Erondu, Adebayo Nehemiah, Suraj Rasaq, Mary Ajayi, Bola Akanle, Olaleye Oladipo, Itunuoluwa Isewon, Marion Adebiyi, Jelili Oyelade, Ezekiel Adebiyi

**Affiliations:** Covenant University Bioinformatics Research (CUBRe), Covenant University, Ota, Ogun State 112104, Nigeria; Covenant University Bioinformatics Research (CUBRe), Covenant University, Ota, Ogun State 112104, Nigeria; South African National Bioinformatics Institute, Life Sciences Building, University of Western Cape, Cape Town 7535, Republic of South Africa; South African National Bioinformatics Institute, Life Sciences Building, University of Western Cape, Cape Town 7535, Republic of South Africa; Covenant University Bioinformatics Research (CUBRe), Covenant University, Ota, Ogun State 112104, Nigeria; Covenant University Bioinformatics Research (CUBRe), Covenant University, Ota, Ogun State 112104, Nigeria; Laboratory of Biotechnology, Environment, Agri-food and Health, Faculty of Sciences Dhar El Mahraz, Sidi Mohammed Ben Abdellah University, Fez 30000, Morocco; Covenant University Bioinformatics Research (CUBRe), Covenant University, Ota, Ogun State 112104, Nigeria; Department of Biological Sciences, Covenant University, Ota, Ogun State 112104, Nigeria; Covenant University Bioinformatics Research (CUBRe), Covenant University, Ota, Ogun State 112104, Nigeria; Department of Computer & Information Sciences, Covenant University, Ota, Ogun State 112104, Nigeria; African Institute for Mathematical Sciences (AIMS), Muizenberg, Cape Town 7945, South Africa; Department of Computer Science, Landmark University, Omu-Aran, Kwara State 251103, Nigeria; Department of Computer Science, Landmark University, Omu-Aran, Kwara State 251103, Nigeria; Department of Computer Science, Landmark University, Omu-Aran, Kwara State 251103, Nigeria; Department of Computer Science, Landmark University, Omu-Aran, Kwara State 251103, Nigeria; Covenant University Bioinformatics Research (CUBRe), Covenant University, Ota, Ogun State 112104, Nigeria; Center for System and Information Services, Covenant University, Ota, Ogun State 112104, Nigeria; Covenant Applied Informatics and Communication Africa Center of Excellence (CApIC-ACE), Covenant University, Ota, Ogun State 112104, Nigeria; Covenant University Bioinformatics Research (CUBRe), Covenant University, Ota, Ogun State 112104, Nigeria; Center for System and Information Services, Covenant University, Ota, Ogun State 112104, Nigeria; Covenant Applied Informatics and Communication Africa Center of Excellence (CApIC-ACE), Covenant University, Ota, Ogun State 112104, Nigeria; Covenant University Bioinformatics Research (CUBRe), Covenant University, Ota, Ogun State 112104, Nigeria; Department of Computer & Information Sciences, Covenant University, Ota, Ogun State 112104, Nigeria; Covenant Applied Informatics and Communication Africa Center of Excellence (CApIC-ACE), Covenant University, Ota, Ogun State 112104, Nigeria; Covenant University Bioinformatics Research (CUBRe), Covenant University, Ota, Ogun State 112104, Nigeria; Department of Computer Science, Landmark University, Omu-Aran, Kwara State 251103, Nigeria; Covenant Applied Informatics and Communication Africa Center of Excellence (CApIC-ACE), Covenant University, Ota, Ogun State 112104, Nigeria; Covenant University Bioinformatics Research (CUBRe), Covenant University, Ota, Ogun State 112104, Nigeria; Department of Computer & Information Sciences, Covenant University, Ota, Ogun State 112104, Nigeria; Covenant Applied Informatics and Communication Africa Center of Excellence (CApIC-ACE), Covenant University, Ota, Ogun State 112104, Nigeria; Covenant University Bioinformatics Research (CUBRe), Covenant University, Ota, Ogun State 112104, Nigeria; Department of Computer & Information Sciences, Covenant University, Ota, Ogun State 112104, Nigeria; Covenant Applied Informatics and Communication Africa Center of Excellence (CApIC-ACE), Covenant University, Ota, Ogun State 112104, Nigeria; Applied Bioinformatics Division, German Cancer Research Center (DKFZ), Heidelberg 69120, Germany

## Abstract

**Motivation:**

Post-genome-wide association studies (pGWAS) analysis is designed to decipher the functional consequences of significant single-nucleotide polymorphisms (SNPs) in the era of GWAS. This can be translated into research insights and clinical benefits such as the effectiveness of strategies for disease screening, treatment and prevention. However, the setup of pGWAS (pGWAS) tools can be quite complicated, and it mostly requires big data. The challenge however is, scientists are required to have sufficient experience with several of these technically complex and complicated tools in order to complete the pGWAS analysis.

**Results:**

We present SysBiolPGWAS, a pGWAS web application that provides a comprehensive functionality for biologists and non-bioinformaticians to conduct several pGWAS analyses to overcome the above challenges. It provides unique functionalities for analysis involving multi-omics datasets and visualization using various bioinformatics tools. SysBiolPGWAS provides access to individual pGWAS tools and a novel custom pGWAS pipeline that integrates several individual pGWAS tools and data. The SysBiolPGWAS app was developed to be a one-stop shop for pGWAS analysis. It targets researchers in the area of the human genome and performs its analysis mainly in the autosomal chromosomes.

**Availability and implementation:**

SysBiolPGWAS web app was developed using JavaScript/TypeScript web frameworks and is available at: https://spgwas.waslitbre.org/. All codes are available in this GitHub repository https://github.com/covenant-university-bioinformatics.

## 1 Introduction

Genome-wide association studies (GWAS) analyses are widely used to report statistically significant genomic variants associated with a particular genetic trait or phenotype (Tam *et al.*, 2019). However, in most cases, researchers are interested in understanding underlying molecular and biological functions that are triggered by these significant variants. Therefore, post-GWAS (pGWAS) analysis is required to address or interpret any GWAS findings (Gallagher and Chen-Plotkin, 2018). Though pGWAS analysis is critical for understanding the genetic mechanisms underlying many traits, it is challenging to perform pGWAS analysis for researchers with limited bioinformatics skills. These challenges are due to the complexity of installing some pGWAS tools, complex command line parameters, or the amount of Genomic data required for the pGWAS analysis. To facilitate pGWAS research, particularly for African bioinformatics and biomedical researchers due to the limited computing resources to store the huge annotated files and reference panels, we built SysBiol pGWAS as a web-based pGWAS tool.

## 2. SysBioPGWAS software

### 2.1. Input and data format

The input file for the SysBiolPGWAS pipeline is a GWAS summary file. A typical GWAS summary file contains nine fields which are: single-nucleotide polymorphisms (SNPs) ID, chromosome, genomic position, reference allele, alternative allele, beta score (effect size), standard error, z-score (summary statistics for SNP association with phenotype) and *P*-value. We used the standard GWAS summary files as described by Buniello *et al.* (2019) and MacArthur *et al.* (2021).

### 2.2 Individual tools and pipelines

**Fig. 1.**
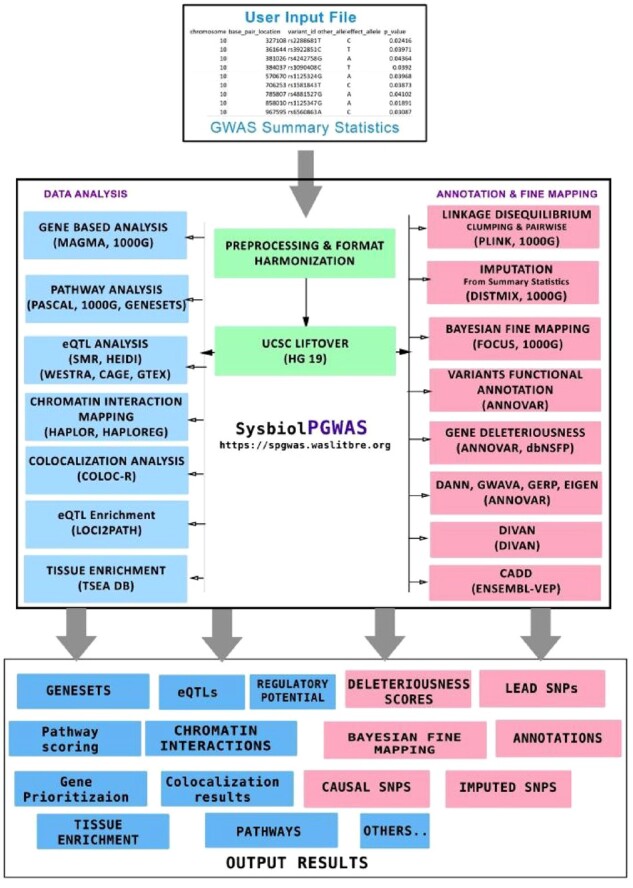
SysBiolPGWAS pipelines and architecture, inputs and outputs. The rectangles in the center of the middle box show the preprocessing pipeline step consisting of cleaning up the input data and lifting over coordinates if needed. The rectangles to the right of the middle box indicate the group of tools that perform annotation of SNPs (detecting the type of SNPs) and fine-mapping (finding the causal SNPs) analysis. The rectangles to the left of the middle box show tools that perform omics based analysis

SysBiolPGWAS provides direct access to several individual pGWAS tool pipelines. These tools are shown in Figure 1. The preprocessing pipeline step consists of cleaning up the input data; and utilizing the University of California, Santa Cruz (UCSC) LiftOver if needed (Haeussler *et al.*, 2019). UCSC LiftOver is a major tool that is used for converting genomic coordinates between different assemblies. This tool is provided as a web-based tool hosted at the University of California, Santa Cruz (UCSC) Genome Browser (https://genome.ucsc.edu/cgi-bin/hgLiftOver). It is also available as a standalone tool (Luu *et al.*, 2020).

The annotation pipeline combines four tools, which are Annovar tool (Wang *et al.*, 2010), Ensembl Variant Effect Predictor (VEP) version 107 (McLaren *et al.*, 2016), the Disease-specific Variant ANnotation tool (DIVAN) (Chen *et al.*, 2016) and deTS software (Pei *et al.*, 2019). The algorithm underlying these annotation tools is to determine what type of variant is being run and to assign an annotation score to the variants based on the source databases. Annovar tool is used to perform several functional scoring of variants. These functional scoring include the genome-wide annotation of variants that supports prioritization of non-coding variants by integrating various genomic and epigenomic annotations (GWAVA) (Ritchie *et al.*, 2014), the functional prediction score generated by deep learning (DANN) (Quang *et al.*, 2015), the functional prediction scores for mutations based on selective constraints across the human genome (GERP++) (Davydov *et al.*, 2010; Cooper *et al.*, 2005) and the spectral approach integrating functional genomic annotations for coding and non-coding variants (EIGEN) (Ionita-Laza *et al.*, 2016). Also, Annovar tool is used for performing SNPs functional annotations and gene deleteriousness based on the dbNSFP database (Liu *et al.*, 2020). On the other hand, Ensembl Variant Effect Predictor is used to perform functional prediction of variants using the Combined Annotation Dependent Depletion score (CADD) (Kircher *et al.*, 2014; Rentzsch *et al.*, 2019). DIVAN tool is used for performing tissue-specific scoring for 45 different diseases/traits. In addition, SysBiolPGWAS performs tissue-specific enrichment analysis for a list of genes that are associated with the variants using deTS (Pei *et al.*, 2019).

The estimating SNPs casualty pipeline step consists of fine-mapping and SNPs clumping. SysBiolPGWAS performs probabilistic fine-mapping, i.e. applied the Bayesian fine-mapping approach, using Fine-mapping Of CaUsal gene Sets software (Mancuso *et al.*, 2019). Bayesian fine-mapping utilizes the statistical Bayes framework to estimate the probability of a given variant being a causal variant, i.e. estimating the Bayes factor (BF) (Wang and Huang, 2022). Such BF can be estimated from GWAS summary statistics such as variant *P*-value and its standard error of the effect even without accessing the individual-level genotype data (Mancuso *et al.*, 2019). Approximation BF from GWAS summary report approximates Bayes factor (Wakefield, 2007, 2009). Maller *et al.* (2012) suggested estimating the probability of variant causality as a posterior inclusion probability (PIP) by using a simplified version of the Bayes model. Moreover, Maller *et al.* (2012) provided a method to estimate the smallest number of variants that can sum up to a predefined PIP threshold value and called it as the credible set. For detailed mathematical equations for the Bayesian fine mapping, refer to Wang and Huang (2022).

SysBiolPGWAS can select causal variants based on the linkage disequilibrium information in 1000 genomes using the clumping method of PLINK software. The process of variant clumping reports iteratively the most significant variant in the defined LD regions across the genome (Choi *et al.*, 2020; Privé *et al.*, 2019). In each LD region, the most significant variant, i.e. the SNP with the smallest *P*-value, is called the lead variant. However, the approach of choosing the lead variants is limited by the biological fact that the leads are not always considered as the causal variants (Schaid *et al.*, 2018).

The step of omic-based analysis and reporting includes five types of analysis, which are (i) gene-level analysis, (ii) pathways analysis, (iii) eQTL analysis, (iv) chromatin interaction mapping [using Position Weight Matrices (PWM)] analysis and (v) colocalization analysis. SysBiolPGWAS uses MAGMA (Multi-marker Analysis of GenoMic Annotation) (de Leeuw *et al.*, 2015) for performing gene-level analysis. The algorithm underlying MAGMA’s gene level is based on the statistical regression approach and utilizing LD information to detect marker effects. SysBiolPGWAS pathways analysis using the Pathway Scoring Algorithm is implemented in Pascal software (Lamparter *et al.*, 2016).

SysBiolPGWAS performs eQTL analysis by incorporating the GTEx multi-tissue eQTL information, which is very useful to understand the biological machinery underlying the variants in GWAS summary. The eQTL analysis can be performed using SMR and HEIDI (Wu *et al.*, 2018), and Loci2Path tool (Xu *et al.*, 2020). For the chromatin interaction mapping (via PWM) analysis, SysBiolPGWAS uses HaploR R package (Zhbannikov *et al.*, 2017) to query HaploRegDB (Ward and Kellis, 2016) and RegulomeDB (Boyle *et al.*, 2012). To perform colocalization analysis, SysBiolPGWAS uses coloc R package (Wallace, 2021).

The interpretation of several predicted variants in a GWAS to functional mechanisms is faced with several challenges identified by Gallagher and Chen-Plotkin (2018), a few of which include: (i) association of an SNP with a phenotype does not give sufficient information about the actual causal SNP or causal gene of that phenotype. (ii) Ninety percent (90%) of SNPs identified in a GWAS fall into a non-protein coding region (intergenic or intronic) which are very far from any known nearest gene. (iii) These SNPs that fall in non-coding regions may be enriched in putative regions of cis-regulatory elements (enhancers, silencers and promoters), however, because of the complex nature of regulation, it might be hard to associate these noncoding cis-regulatory elements (CREs) to correct target genes. We chose all these crop of tools in this study to help tackle these challenges. We also selected appropriate tools that help annotate variants in protein-coding and non-coding regions. In summary, each tool in the individual pipelines provides a unique pGWAS analysis to assist biologists in performing pGWAS analysis in one place, seamlessly overcoming the complex challenges and obtaining the results expeditiously.

### 2.3 Customized pipeline

This pipeline integrates several pGWAS tools and allows users to execute several of these tools in a single run. It should be noted that this pipeline performs pGWAS analysis only on the potential lead SNPs. First, we perform SNPs clumping to find lead SNPs, then we do gene-based analysis, followed by pathways analysis, eQTL analysis, SNPs annotations, deleteriousness and regulation analysis. We also execute Bayesian fine-mapping to report the probability of each SNP to be casual.

## 3 Conclusion

In conclusion, SysBiolPGWAS provides several pipelines consisting of multiple pGWAS tools with the current state-of-the-art annotation tools to perform complete pGWAS analysis and visualization for all users, especially scientists who are not command line or statistically inclined. This is the first version of the tool, and we plan to extend SysBiolPGWAS to include more comprehensive OMICS, further GWAS and pGWAS analysis by including more pGWAS resources and tools. Online tutorials are available in the tutorial navigation link drop down section at https://spgwas.waslitbre.org/.
